# Genome-wide DNA methylation in Human Brain and Molecular Pathologies associated with Suicide Among Depressed Patients

**DOI:** 10.1192/j.eurpsy.2025.512

**Published:** 2025-08-26

**Authors:** Y. Dwivedi

**Affiliations:** Psychiatry, University of Aalabam at Birmingha, Birmingham, United States

## Abstract

**Introduction:**

Major depressive disorder (MDD) is the single most important risk factor for suicide. Interestingly, even with a high prevalence of suicidality, not all MDD patients develop suicidal thoughts or complete suicide. Thus, it is critical to examine the risk factors that can distinguish suicidality among MDD patients.

**Objectives:**

It has been hypothesized that epigenetic marks, such as DNA methylation, can be influenced by the environment, which may play a critical role in developing depression and suicidal behavior. The present study examined genome-wide DNA methylation in the prefrontal cortex of depressed suicide (DS), depressed non-suicide (DNS), and nonpsychiatric control subjects.

**Methods:**

Genome-wide DNA methylation was examined in the prefrontal cortex of age- and sex-matched depressed suicide, depressed non-suicide, and nonpsychiatric control subjects using 850K Infinium Methylation EPIC BeadChip. The methylation β values were generated based on normalized signal intensities and background subtraction using negative control probes and were derived as the ratio of methylation probe intensity to the overall intensity. The hyper and hypo-methylated sites were also mapped across 22 autosomes using PhenoGram Plot. The significantly differentially methylated gene lists were used to determine the functional enrichment of genes for ontological clustering and pathway analysis.

**Results:**

The chromosome-wise methylation sites and mapping of methylated sites based on the number of CpG content and their relative distribution from specific landmark regions of genes identified 32958 methylation sites 12574 genes in NC vs. all MDD subjects, 30852 methylation sites across 12019 genes in NC vs. DNS, 41648 methylation sites across 13941 genes in NC vs. DS, and 49848 methylation sites across 15015 genes in DNS vs. DS groups. A comparison of methylation sites showed 33129 unique methylation sites and 5451 genes in the DNS group compared to the DS group. Functional analysis suggested Oxytocin, GABA, VGFA, TNFA, and MTOR pathways associated with suicide in the MDD group.

**Conclusions:**

Our data show a discrete pattern of DNA methylation, the genomic distribution of differentially methylated sites, gene enrichment, and pathways in MDD subjects who died by suicide compared to non-suicide MDD subjects and suggest that epigenetic DNA modifications could be used to distinguish suicidality in MDD patients.

**Disclosure of Interest:**

None Declared

